# Introducing Selectivity on Carbonaceous Material: Removing Noble Salts, Au^3+^, and Ag^+^ from Aqueous Media by Nanodiamonds Functionalized with Squaramides

**DOI:** 10.3390/ma13051086

**Published:** 2020-02-29

**Authors:** M. Susana Gutiérrez, Kenia A. López, Jeroni Morey, M. Nieves Piña

**Affiliations:** Department of Chemistry, University of the Balearic Islands, Cra. de Valldemossa, Km. 7.5, 07122 Palma de Mallorca, Balearic Islands, Spain; dominika71@hotmail.com (M.S.G.); kenia1410@hotmail.com (K.A.L.)

**Keywords:** nanodiamonds, squaramides, silver ion, gold ion

## Abstract

Nanodiamonds coated with dopamine−squaramide compounds have been prepared by a calcination/esterification synthetic process, which improves the efficiency of this carbonaceous material with respect to non-functionalized nanodiamonds. The modified nanodiamonds show excellent selective coordination of Ag^+^ and Au^3+^ cations in a Cd^2+^, Co^2+^, Cr^3+^, Cu^2+^, Pb^2+^, and Zn^2+^ mixture in water. The coordination capacity of the carbonyl squaramide groups with the silver and gold cation is based on purely electrostatic cation−dipole interactions. Overall, it is demonstrated that the conjunction between the nanodiamonds and the organic receptor improves the selectivity of the material toward noble cations.

## 1. Introduction

The use of inorganic materials, such as silicates [1], clays [2], metal oxides [3], and carbon-based materials, for the removal [4,5] by adsorption of impurities in aqueous media is a traditional methodology used in water purification. The best example is the activated carbon used in water and air purification filters. This material has unique physical−chemical properties, such as being affordable, thermally stable, and chemically inert. Its high specific surface area and large pore size make it, to date, the best industrial adsorbent for this purpose. However, one of the major drawbacks of activated carbon is its lack of selectivity, which hinders its use in more specialized tasks.

Nanodiamonds (NDs) are a crystalline form of carbon first described in 1963 [6,7]. NDs are inert, photostable, and biocompatible nanoparticles based on carbon that present a homogeneous, well-defined morphology and are not bio-accumulative. This material gives good dispersion in water, with a size between 2 nm and 10 nm, good thermal conductivity, and a high refractive index (n = 2.42). Historically, the most prominent applications of these nanomaterials have been biological due to their biocompatibility, such as in biosensors and drug delivery [8,9]. However, the nanodiamonds can be used in environmental monitoring [10]. Nanodiamond-containing polyethyleneimine hybrid materials have been used in lead adsorption from aqueous media [11]. Especially, the nanodiamonds can be surface-grafted with rare-earth metal ions like Ce, Pr, Sm, Eu, Gd, Dy, Ho, Tm, and Eu [12].

The surface properties of NDs can be tuned during the preparation method, usually by detonation (dND) in a closed chamber in the presence of air and concentrated mineral acids for purification [13]. With this method, mechanically robust nanoparticles are obtained, and their most relevant chemical characteristic is the presence of oxygenated functional groups on their surface, such as carboxylic acids, lactones, ethers, ketones, and alcohols. Through a calcination process, these functional groups are mostly transformed into carboxylic groups, also eliminating other residues and impurities, such as graphite and amorphous carbon residues generated during preparation. This thermal treatment is sufficient to achieve good anchorage points that allow the functionalization of the ND surface with different organic receptors, which improve and optimize the material for use in different technological applications [14,15].

The magnetic iron oxide nanoparticles coated with squaramide−dopamine-derived nanoparticles have demonstrated a high capacity for the complexation of heavy metals and transition metals, such as Cr^3+^, Pb^2+^, Hg^2+^, Zn^2+^, Cd^2+^, Co^2+^, or Cu^2+^ [16], as well as salts derived from Ag^+^ and Au^3+^ [17] in aqueous media. Due to their low diffusion and low toxicity, it is considered that the environmental and health impacts of salts derived from Ag^+^ and Au^3+^ in water are minimal. However, in certain circumstances, especially where electronic components are manufactured, there is a possibility that their concentration in a specific aqueous environment would be significant, so they can be considered as emerging pollutants, produced by electronic industries and known as e-wastes [18]. These pollutants are currently given special attention for health and/or economic reasons, which is why their removal and recovery from the environment are necessary. The development of new selective technologies for identification, capture, and disposal, using adsorbent and affordable materials, is necessary for this.

**Figure 1:**
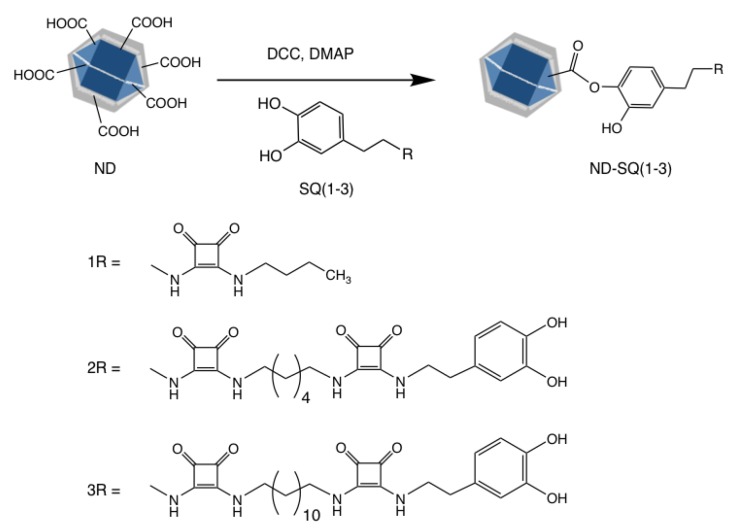
Scheme of the preparation of hybrid nanodiamond squaramide derivatives used in this study (ND-SQ(1−3)).

The present study describes the functionalization of NDs synthesized by detonation, with supramolecular receptors that optimize and enhance the adsorption capacity of metal ions, thus forming a super-functional hybrid tandem (Figure 1).

The strategy used involves a direct esterification reaction on the surface of the NDs, using the COOH groups present to react with one of the phenolic groups of the catechol units of the dopamine−squaramide moiety.

## 2. Materials and Methods

Reactions were carried out in oven-dried glassware under an atmosphere of argon, unless otherwise indicated. Diamond nanopowder (spherical), < 10 nm particle size (TEM), ≥ 95% traces metals basis, was purchased from Sigma-Aldrich (Merck Life Science S.L.U., Madrid, Spain). All reagents and solvents were purchased from Sigma-Aldrich or Scharlab (Scharlab, S. L., Barcelona, Spain). FT-IR analysis of samples was performed on a Bruker Tensor 27 instrument (Bruker Española, Madrid, Spain) by mixing dried nanodiamond–dopamine–squaramide powders into KBr pellets. Thermogravimetric analysis (TGA) was performed on an SDT Q600 TA (TA Instruments, New Castle, DE, U.S.A.) in Al_2_O_3_ crucibles. Dried samples (typically 7−8 mg) were initially held at 40 °C for 10 min before heating to 800 °C at a rate of 10 °C min^−1^ in an N_2_ atmosphere. ^1^H and ^13^C NMR spectra were recorded on a Bruker Advance III Spectrometer (Bruker Española S.A., Madrid, Spain) at 300 and 75 MHz at 23 °C, respectively. ^13^C NMR solid-state spectra were recorded on a Bruker Advance III Spectrometer at 600 MHz at 23 °C. Chemical shifts are reported as parts per million (δ ppm) referenced to the residual protium signal of deuterated solvents. Electrospray mass spectra (HRMS-ESI) were recorded with a Micromass Autospec 3000 spectrometer (Mettler-Toledo, Barcelona, Spain) provided with an electrospray module. The specific surface area was determined by the Brunauer−Emmett−Teller (BET) method; gas adsorption measurements were performed in an N_2_ atmosphere at 77 K on a Micrometrics Tristar II Surface Area and Porosity Analyser (Micrometrics Instrument Corp., GA, U.S.A.). Analysis by an inductively coupled plasma-optical emission spectrometer (ICP-OES) was performed on a PERKIN ELMER Optima 5300 DV instrument (Thermo Fisher Scientific, Waltham, MA, U.S.A.). Transmission electron microscopy (TEM) was achieved using a Philips JEOL JEM 2100F (Jeol, Zurich, Switzerland). The zeta potential was measured with a Malvern Zetasizer Nano ZS (Malvern Panalytical, Malvern, United Kingdom) at 25 °C. 

## 3. Results

### 3.1. Synthesis of 3-(Butylamino)-4-((3,4-dihydroxyphenethyl)amino)cyclobut-3-ene-1,2-dione. (1R)

Dopamine hydrochloride was dissolved in anhydrous methanol (5 mmol, 25 mL) followed by the addition of Na_2_CO_3_ (400 mg) and Na_2_S_2_O_4_ (200 mg). The pH of the mixture was adjusted with NaOH 1 M until approximately 8. Subsequently, 5 mmol of 3-(butylamino)-4-ethoxycyclobut-3-ene-1,2-dione dissolved in 15 mL of methanol was added dropwise; the reaction mixture was stirred for 12 h under Ar atmosphere and protected from sunlight. Finally, the solvent was completely evaporated, and the product was washed with Et_2_O/CH_2_Cl_2_ (5:1; 5 × 15 mL). (0.75 g, 49%). Mp = 213 °C. ^1^H-RMN (DMSO-d_6_) δ: 8.57 (br, 2H); 7.80 (br, 2NH); 6.61 (m, 2H); 6.46 (m, 1H); 3.63 (t, 2H); 3.47 (t, 2H); 2.64 (t, 2H); 1.46 (m, 2H); 1.31 (m, 2H); 0.88 (t, 3H) ppm. ^13^C-RMN (DMSO-d_6_) δ: 182.67, 168.24, 145.44, 144.14, 129.83, 119.94, 116.71, 116.03, 45.53, 43.50, 36.94, 33.30, 19.51, 14.05 ppm. FTIR (KBr): 3170, 2959, 1799, 1649, 1582, 1432, 1357, 1298, 1146, 1112, 951, 815, 757, and 614 cm^−1^. HRMS-ES(+) found *m*/*z* 327.1324 [M + Na]^+^, C_16_H_20_N_2_O_4_Na requires 327.1321.

### 3.2. Synthesis of 4,4’-(Hexane-1,6-diylbis(azanediyl))bis(3-((3,4-dihydroxyphenethyl)amino)cyclobut-3-ene-1,2-dione) and 4,4’-(Dodecane 1,12-diylbis(azanediyl))bis(3-((3,4-dihydroxyphenethyl)amino)cyclobut-3-ene-1,2 dione). (2R and 3R)

Dopamine hydrochloride was dissolved in anhydrous methanol (2 mmol, 25 mL) followed by the addition of Na_2_CO_3_ (100 mg) and Na_2_S_2_O_4_ (50 mg). The pH of the mixture was adjusted with NaOH 1 M until approximately 8, and stirred for 2 h. Next, 1 mmol of 3-((3,4-dihydroxyphenethyl)amino)-4-((6-((2-ethoxy-3,4-dioxocyclobut-1-en-1-yl)amino)hexyl)amino)cyclobut-3-ene-1,2-dione or 3-((3,4-dihydroxyphenethyl)amino)-4-((12-((2-ethoxy-3,4-dioxocyclobut-1-en-1-yl)amino)dodecyl)amino)cyclobut-3-ene-1,2-dione dissolved in 15 mL of methanol was added dropwise. The reaction mixture was stirred for 12 h under Ar atmosphere and protected from sunlight. Finally, the mixture was filtrated, and the solvent was completely evaporated. The final product was recrystallized in acetonitrile. 

2R: Yield 66%. Mp > 200 °C. ^1^H-RMN: (DMSO-d_6_) δ: 7.78 (br, 4H), 6.64 (m, 4H), 6.47 (m, 4H), 3.63, (t, 4H), 2.85 (t, 2H), 2.64 (t, 4H), 1.50 (m, 4H), 1.31 (m, 4H), and 1.11 (m, 2H) ppm. ^13^C (DMSO-d6) δ: 182.53; 182.42; 167.97; 145.32; 143.89; 129.40; 119.58; 116.31; 115.70; 48.81; 45.12; 43.38; 40.35; 40.08; 38.96; 38.68; 36.62; 30.84 and 25.62 ppm. FTIR (KBr): 3452, 3174, 2924, 2850, 1800, 1644, 1585, 1433, 1355, 1196, 1109, 754 y 617 cm^−1^. MS (Matrix-assisted laser desorption/ionization time-of-flight mass spectrometer) (MALDI-TOF) found *m*/*z* 601.2269 [M + Na]^+^, C_30_H_34_N_4_O_8_Na requires 601.2274.

3R: Yield 76%. Mp > 200 °C. ^1^H-RMN: (DMSO-d_6_) δ: 8.80 (s, 2H), 8.72 (s, 2H), 7.34 (br, 4H), 6.60 (m, 4H), 6.44 (m, 2H), 3.36 (t, 4H), 2.64 (t, 4H), 1.48 (t, 4H) y 1.24 (m, 20H) ppm. ^13^C (DMSO-d_6_) δ: 182.32; 167.79; 167.11; 145.15; 143.72; 129.20; 119.35; 116.12; 115.50; 70.82; 44.91; 43.22; 40.35; 40.07; 38.97; 38.69; 37.62; 36.52; 30.73; 28.97; 28.60 and 25.82 ppm. FTIR (KBr): 3452, 3171, 2913, 2847, 1799, 1650, 1491, 1450, 1352, 1298, 1200, 1147, 1111, 951, 813, 616 y 542 cm^−1^. MS (MALDI-TOF) found *m*/*z* 663,3388 [M]^+^, C_36_H_46_N_4_O_8_ requires 662,3316.

### 3.3. Modification of ND

The NDs were calcinated at 450 °C in a muffle furnace for 4 h. Then, the NDs were cleaned with Milli-Q water (2 × 100 mL) in an ultrasonic bath and, finally, dried perfectly with a vacuum pump.

### 3.4. ND Nanoparticles Were Functionalized with Dopamine–Squaramide (1R-3R)

The calcined NDs (8.3 mmol) were suspended in 25 mL anhydrous DMF, and 4-dimethylaminopyridine (DMAP) was then added (0.4 mmol). The mixture was stirred for 30 min at 45 °C and, subsequently, 0.35 mmol of squaramide receptor (SQ1-SQ3) and 0.5 mmol of *N*,*N’*-dicyclohexylcarbodiimide (DDC) were added. The mixture was boiled and stirred under Ar atmosphere overnight. Functionalized ND was centrifuged at 5000 rpm and decanted, washed with Milli-Q water (2 × 25 mL), and finally dried with a vacuum pump.

### 3.5. Metallic Cations Quantification

Solutions of Ag^+^, Au^3+^, Co^2+^, Cr^3+^, Cu^2+^, Pb^2+^, Cd^2+^, and Zn^2+^ of 100 ppm in concentration were prepared in HNO_3_ (2% v/v) by dilution from commercial standards of 1000 ppm. The test samples were prepared using specific amounts of each metal solution, and each type of ND, directly in ICP test tubes. The ND samples (1 mg of each) were dispersed in HNO_3_ (2% v/v) gauging the sample to 10 mL to obtain a final concentration of 5 ppm of each metal, per sample, including calcined NDs as a reference pattern. Samples were constantly shaken for 3 h and then filtered with 0.22 μm PVDF filters, and the remaining solutions were analyzed by inductively coupled plasma-optical emission spectrometry (ICP-OES). For the competition and selectivity assays, a mix of metals discussed above, with an equal concentration of each (1 ppm) in HNO_3_ (2% v/v), was mixed with the different NDs (1 mg of each) and shaken for 3 h, and then filtered with 0.22 μm PVDF filters to analyze the remaining liquid. The ICP-OES assays were performed on a Perkin Elmer Optima 5300 DV.

### 3.6. 13.C CP-MAS NMR Experiments

These experiments were performed on a Bruker Advance 600 MHz NMR spectrometer using a 4 mm double resonance broadband MAS probe (HX) at room temperature. Samples were prepared by packing the NDs in a 4 mm zirconia rotor. A spinning speed of 13 kHz was employed for all measurements. Data were collected using a cross-polarization sequence under Hartmann−Hahn conditions with magic angle spinning and dipolar decoupling. A contact time of 1.7 ms and repetition recycle delay of 3 s were used. A spinal-64 decoupling sequence was used, and 15,000 scans were collected for signal averaging. Glycine carbonyl resonance at 175.2 ppm and methylene groups at 62.4 ppm were used as an external standard for chemical shift calibration. The time of the experiment was approximately 18 h.

## 4. Discussion

**Figure 2:**
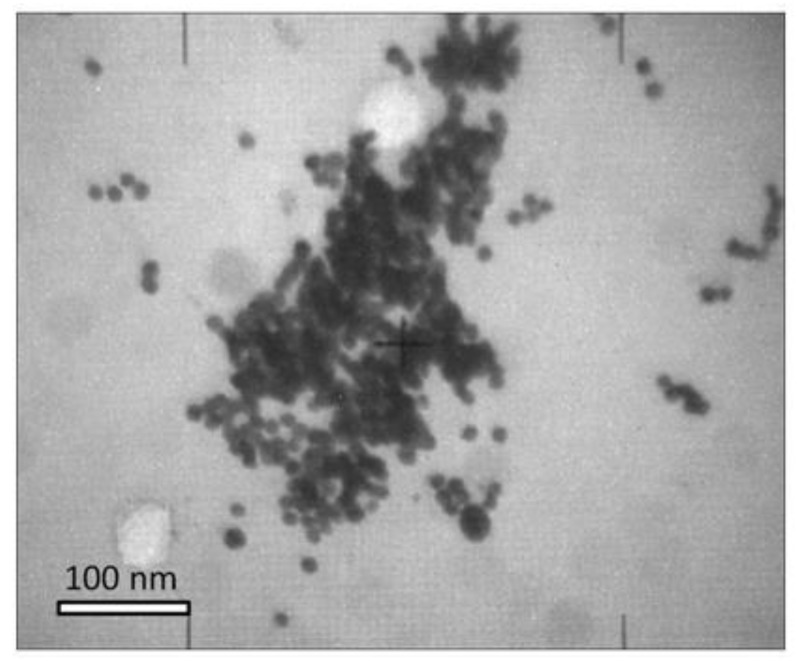
TEM micrograph of nanodiamonds used in this study.

The NDs obtained by the detonation method have a high degree of purity, with a particle size less than 10 nm. Figure 2 shows a photomicrograph performed by TEM; it can be seen that nanodiamonds present a homogeneous morphology. The surface area of the starting ND was 263 m^2^/g determined by the BET method, with an average pore diameter of 18.2 nm and maximum absorption of 788 m^3^/g.

The treatment by calcination at 450 °C for 4 h produces oxidation of the hydroxyl groups on the surface of the nanodiamonds to carboxylic acids. This superficial modification clearly affects the value of the zeta potential of the starting ND. Thus, before calcining the sample, it has a positive value of the zeta potential of +31.7 mV due to the protonation of the superficial amine groups, as well as the presence of graphite residues (sp^2^ carbons). After calcination, the zeta potential changes to a negative value of −27.7 mV, characteristic of the existence of carboxylate groups on the surface. This heat treatment ensures that the most abundant functional groups on the surface of the ND are carboxylate groups.

**Figure 3:**
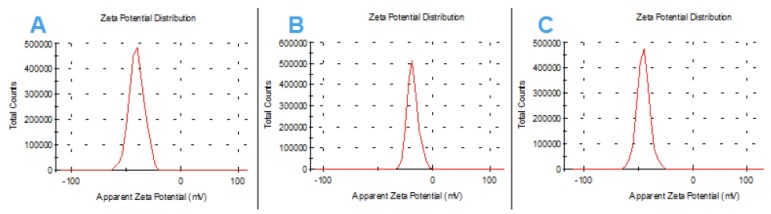
Zeta potential distribution for (**A**) ND-SQ1, (**B**) ND-SQ2, and (**C**) ND-SQ3.

The modification with the dopamine–squarate residues of the surface carboxylates is carried out by esterification, using one of the hydroxyl groups present in the catechol ring of the dopamine–squarate, Figure 1: SQ (1−3). Thus, with the formation of these new covalent bonds, the starting NDs are superficially coated, obtaining three new hybrid materials: ND-SQ (1−3), whose most relevant characteristic, from a supramolecular recognition point of view, is the superficial presence of squaramide residues capable of interacting through the carbonyl groups of the squarate ring with different metal ions present in an aqueous solution. The presence of a final dopamine unit, in the case of functionalization with the ND-SQ2 and ND-SQ3, also provides the possibility of the union of two NDs with the same squaramide–dopamine in a double esterification reaction. This possibility, and that double esterification can be formed on the same ND, is statistically possible. The correct functionalization of the NDs with the dopamine–squaramide residues SQ (1−3) has been verified with several techniques. The hydrodynamic size of each of the functionalized NDs measured by dynamic light scattering (DLS) is 278.7, 484.2, and 1128 nm for ND-SQ (1−3). The observed values of the zeta potential of the three new hybrid materials are all negative. This is due to the superficial presentation of the remaining non-esterified catechol hydroxyl groups, or to the starting surface carboxylate residues on the NDs that have been left without reacting. The values obtained are −40.4, −18.1, and −45.6 mV for ND-SQ (1−3), respectively (Figure 3). 

**Figure 4:**
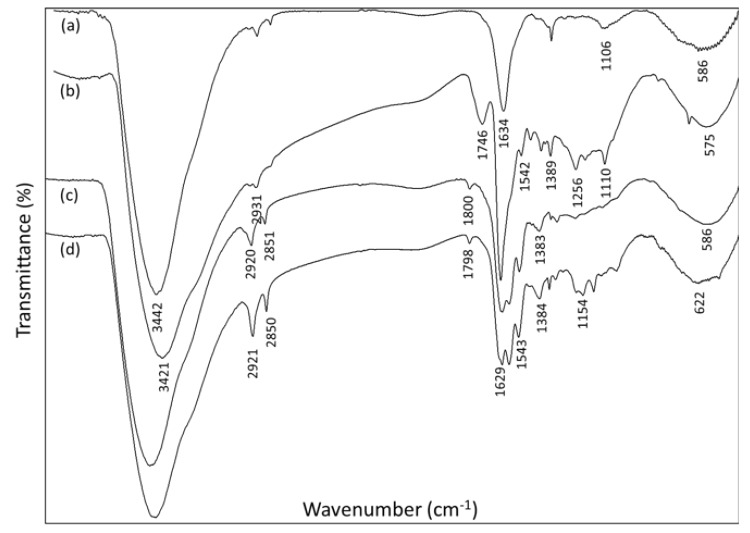
FTIR of (**a**) non-functionalized nanodiamonds, (**b**) ND-SQ1, (**c**) ND-SQ2, and (**d**) ND-SQ3.

Another test that supports the surface functionalization of the ND can be seen in the solid-state FTIR spectra (KBr pellets) of the corresponding ND-SQ hybrid materials (1−3) (Figure 4, FTIR). The FTIR spectrum of previously calcined non-functionalized NDs (Figure 4) is simple and shows few bands. The band at 3442 cm^−1^ corresponds to an intense O−H stretch band; at 1634 cm^−1^, the tension band C=O of the carboxylic acids is observed; and finally, a weak stretch band C−O characteristic of the ND is located at 1106 cm^−1^ [19]. In the FTIR spectra of the three samples of functionalized NDs, the characteristic bands corresponding to the tension band C=O of the squarate ring in 1746, 1800, and 1798 cm^−1^ are observed, respectively. At 1649, 1632, and 1629 cm^−1^, respectively, tension bands C=O of the amide group (amide I band) appear, while 1542, 1546, and 1592 cm^−1^, respectively, correspond to the deformation bands of the N−H bond of the amide group (amide II band).

Finally, bands located at 1389, 1383, and 1384 cm^−1^ can be observed, which correspond to the tension band of the C−O bond of the esters formed.

**Figure 5:**
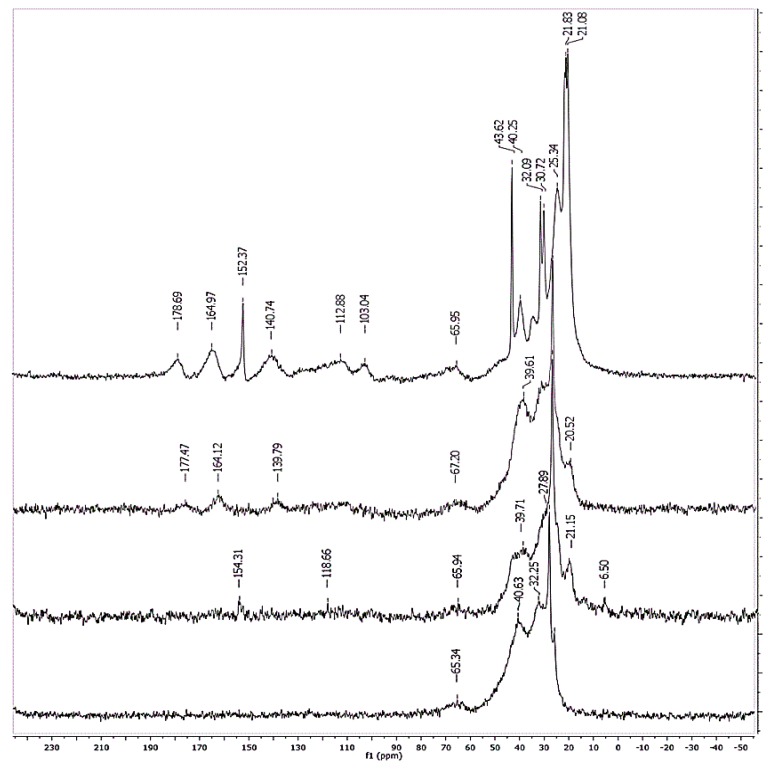
^13^C CP-MAS NMR of NDs: (**a**) Unfunctionalized, (**b**) ND-SQ1, (**c**) ND-SQ2, and (**d**) ND-SQ3.

Likewise, the right modification of the newly synthesized hybrid material has been checked by ^13^C CP-MAS NMR. In Figure 5, ^13^C CP-MAS and ^13^C NMRs obtained for a sample of non-functionalized calcined ND are shown, together with the corresponding ^13^C NMR spectra for the three newly prepared hybrid materials. In the ^13^C CP-MAS corresponding to the non-functionalized NDs, an intense broadband centered at δ = 32.6 and 36.9 ppm is observed, which is attributed to the C sp^3^ of a diamond. The signal with the lowest intensity at δ = 68.6 ppm corresponds to the C of the –OH groups located on the surface.

The ^13^C CP-MAS of the compound ND-SQ3 is coated with the di-squaramide SQ3 (Figure 5d), and the longer one has a better spectral resolution. The signal can be observed due to C=O groups of squaramide at δ = 178.7 ppm, and the overlap of the signal of ester groups and the C=C of the cyclobutene ring of squaramide at δ = 165 ppm. The signals of dopamine aromatic rings are in the frequency range of 100–155 ppm. Finally, the peaks corresponding to −CH_2_−, the resonance of dopamine [20], and the methylene chain linked to the squaramide units appear in the frequency range 20–50 ppm [21,22]. The broad signal at δ = 66 ppm corresponds to the C−OH groups located on the surface of the NDs.

**Figure 6:**
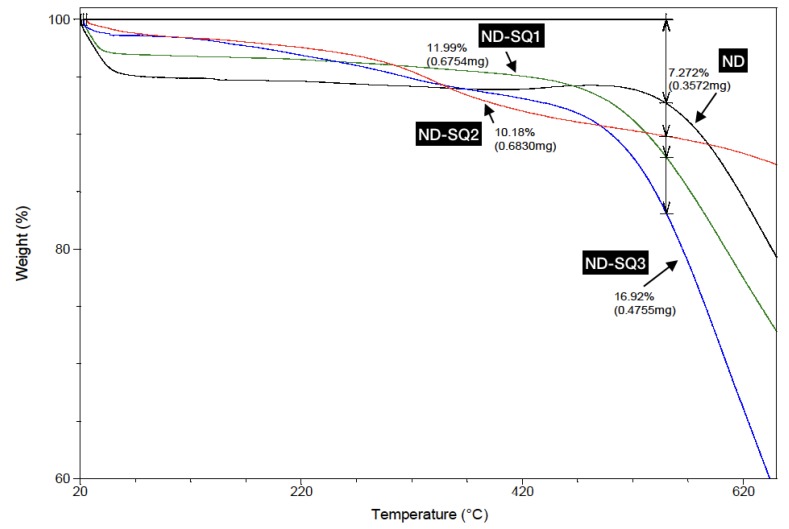
Thermogravimetric analysis (TGA) for ND, ND-SQ1, ND-SQ2, and ND-SQ3.

The thermogravimetric analyses, TGAs, carried out in the temperature range between 20 °C and 450 °C, show, in all cases, a single weight loss, corresponding to the elimination of the superficial COOH and OH groups (Figure 6). The unfunctionalized NDs only lose 7.3% by weight, while in the functionalized ND samples, the loss of recorded mass is somewhat greater, such as 12.0% for ND-SQ1, 10.2% for ND-SQ2, and 16.9% for ND-SQ3.

To evaluate the coordination capacity of the new hybrid nanomaterials with various metal ion salts, typically pollutants, such as Cr^3+^, Pb^2+^, Zn^2+^, Cd^2+^, Co^2+^, and Cu^2+^, as well as with salts derived from Ag^+^ and Au^3+^, two types of experiments were performed. One for studying the retention capacity of each metal salt, individually, by the three hybrid-modified nanodiamonds; the other for evaluating the selectivity of new materials, determining the retention percentage of each metal ion from a multicomponent solution.

In the individual tests, solutions of Ag^+^, Au^3+^, Cd^2+^, Co^2+^, Cr^3+^, Cu^2+^, Pb^2+^, and Zn^2+^ were prepared for each metal, using 2 mL HNO_3_ at a concentration of 100 ppm, adding approximately 1 mg of ND-SQ (1−3), respectively, to each sample. Then, 9.5 mL of 2% *v/v* HNO_3_ was added to reach a final concentration of 5 ppm.

Each sample, of each metal ion, independently, was kept under stirring for 3 h until the adsorption/desorption equilibrium was reached. Subsequently, the suspension was filtered, and the concentration remaining in the solution of each metal ion was determined by inductively coupled plasma (ICP).

**Figure 7:**
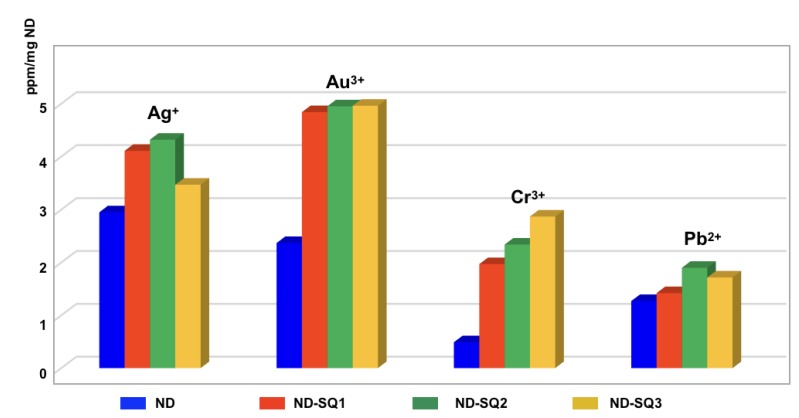
Retention of metal cations by the non-functionalized ND, ND-SQ1, ND-SQ2, and ND-SQ3.

The results obtained are shown in Figure 7, where the concentration (in ppm) of the metal retained per milligram of ND used is represented. In addition, along with these results, the retention obtained using the non-functionalized NDs, as a control, is shown.

Good retention for Ag^+^, Au^3+^, Cr^3+^, and Pb^2+^ is observed, with Au^3+^ being the best-retained metal cation of all, with a retention close to 100% (≈ 5 ppm). For non-functionalized NDs, only 50% of adsorption of Au^3+^ salts is observed.

Like the Au^3+^ salts, the Ag^+^ salts show good retention, registering a maximum value of 4.32 ppm per mg of ND-SQ2, 4.11 ppm for the ND-SQ1, and 3.47 ppm for the ND-SQ3.

Cr^3+^ salts have a maximum retention of 2.87 ppm per milligram of ND-SQ3, and of 2.34 and 1.97 ppm for ND-SQ2 and ND-SQ1, respectively. With Cr^3+^ salts, the non-functionalized NDs have very low retention values.

The salts of Pb^2+^ are retained to a smaller amount with the ND-SQ (1−3), so the best result is obtained with the ND-SQ2 with 1.90 ppm retained for each milligram of nanomaterial. The ND-SQ3 has a retention of 1.72 ppm for each milligram. Finally, ND-SQ1 retains 1.42 ppm per mg of functionalized diamond nanoparticles, which is very close to the control test.

The metal salts of Cd^2+^, Co^2+^, Cu^2+^, and Zn^2+^ show no retention in any case, including the non-functionalized ND.

**Figure 8:**
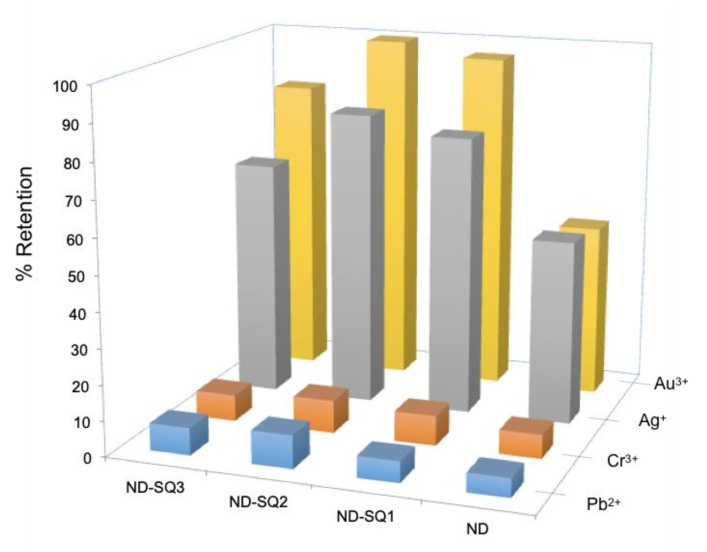
Percentages of retention for different hybrid materials and metal ions in water solution.

For competitive experiments, a mixture of all cations tested in equal concentration (≈ 1 ppm) in 2% *v/v* HNO_3_ was prepared. The solution was kept in contact with approximately 1 mg of each of the three hybrid NDs and the non-functionalized NDs, under stirring for 3 h. Finally, they were filtered, and the remaining concentration of each metal was quantified by ICP. The results, shown in Figure 8, demonstrate the excellent selectivity of the functionalized and non-functionalized NDs for the salts of Au^3+^ and Ag^+^.

The results obtained are consistent with those of the individual tests. The special affinity for Au^3+^ and Ag^+^ prevails over the other cations, almost completely saturating the NDs, as can be seen in Figure 8. The retention of Cr^3+^ and Pb^2+^ decreases drastically as they are displaced by the adsorption of Ag^+^ and Au^3+^.

The ND-SQ2 receptor was identified as the best receptor, with 83.22% retention for Ag^+^ and 99.73% for Au^3+^ for milligrams of material, while for Cr^3+^ and Pb^2+^, very similar levels of retention are observed, reaching only 9.27% and 9.25%, respectively, for each milligram of ND-SQ2.

## 5. Conclusions

**Figure 9:**
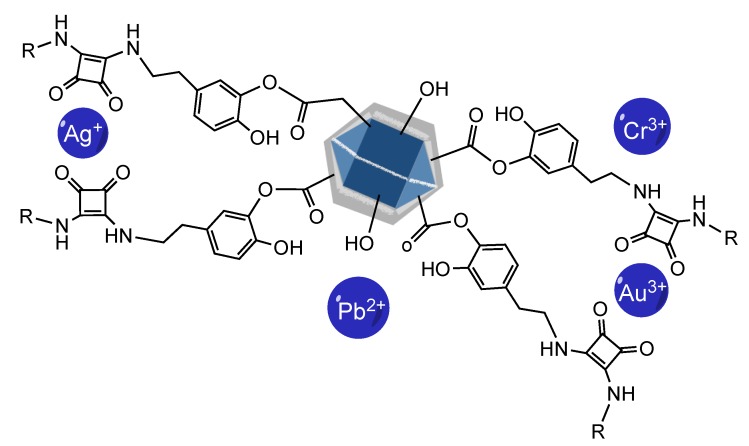
Proposal of the interaction between ND-SQ1 and the Au^3+^ and Ag^+^ ions.

The functionalization of NDs with dopamine–squaramide residues covalently bonded onto their surface has been demonstrated by ^13^C CP-MAS. We show that this coating provides the new materials with greater selectivity and efficiency in the uptake of noble metal salts, such as gold and silver, against a mixture of metal cations in an aqueous medium. This coordination is produced by the electrostatic cation–dipole interaction that is established between the electron pairs of the carbonyl groups of the cyclobutendienone ring of the squaramide and the metal cation. Depending on their surface distribution, in some cases, two receivers can act together on the same ion (Figure 9).

It has also been shown that the NDs are environmentally safe inorganic platforms valid for functionalization with biocompatible molecular receptors, such as SQ1, SQ2, and SQ3. In addition, we demonstrate that the carboxylic acid groups present on the surface of NDs (as in other carbonaceous materials, such as Starbon^®^ [23,24], biochar [25,26], and activated carbon [27]), are an excellent anchor point to increase the selectivity and performance of raw materials.
